# Estrogen Receptor Beta (ERβ) Expression in Colorectal Cancer (CRC)—A Systematic Review and Meta-Analysis

**DOI:** 10.3390/jcm15155924

**Published:** 2026-07-29

**Authors:** Yael Sher, Yaron Niv

**Affiliations:** Adelson Faculty of Medicine, Ariel University, Ariel 40700, Israel; nivy@ariel.ac.il

**Keywords:** CRC, ERβ, systematic review, meta-analysis, gene expression

## Abstract

**Background:** ERβ is an estrogen receptor isoform expressed in the normal human colon and has been proposed to act as a tumor suppressor gene. The loss of ERβ expression has been associated with advanced stages of CRC. **The aim was** to investigate the relationship between ERβ expression and CRC progression, quantifying the difference between tumor tissues and normal controls, and early-stage versus late-stage tumors. **Methods:** English Medical literature searches were conducted using PubMed, Embase, and Google Scholar up to 31 December 2025 according to PRISMA. Case–control studies comparing ERβ expression in CRC biopsy specimens with that in healthy controls were included. A meta-analysis was performed using Comprehensive Meta-analysis (Version 4). Pooled ORs and 95% confidence intervals were calculated using a random-effects model. Heterogeneity was assessed using the Cochrane Q test and the I^2^ statistic. Publication bias was evaluated. **Results:** Six studies, representing 13 sub-studies, were selected according to the inclusion criteria, involving 1183 patients and 1000 **normal mucosa**/tumor early-stage patients. The OR for ERβ expression was 0.211 (95%CI: 0.122–0.363), significantly lower in advanced tumor stages than in normal mucosa and early tumor stages (*p* < 0.0001). Positivity rates were 76.40% in controls and early-stage tumor patients versus 51.31% in tumor patients (all stages). Heterogeneity was high (I^2^ = 78%), but sensitivity analysis confirmed the robustness of the results. Publication bias was not significant. The studies used different methods and positivity cut-off values, with high heterogeneity, and were from only five countries, indicating the possibility of variation in results across other geographical areas or ethnic groups. **Conclusions:** ERβ expression is significantly lower in CRC tissues than in healthy controls and progressively decreases as the disease progresses. This finding underscores ERβ’s potential role as a prognostic factor and highlights the viability of ERβ activation as a novel therapeutic strategy.

## 1. Introduction

Estrogen is a pleiotropic steroid hormone that exerts pivotal roles across various tissues, including the male and female reproductive tract, the skeletal, nervous and immune systems, and notably, the gastrointestinal tract [1]. Estrogen modulates processes such as tissue development, differentiation, and growth, primarily by binding to estrogen receptors (ERs). These receptors, classified into estrogen receptor alpha (ERα) and estrogen receptor beta (ERβ), mediate their influence by activating downstream signaling pathways and modulating the expression of target genes after binding to DNA [1]. ERα was first characterized in 1985, while ERβ was identified in 1996 [2].

ERβ is a member of the nuclear receptor superfamily and is the predominant estrogen receptor expressed in the normal colonic mucosa [3]. Substantial preclinical evidence supports the hypothesis that ERβ functions as a potent tumor suppressor gene across various gastrointestinal cancers, including colorectal, gastric, esophageal, and pancreatic cancers [3]. Loss of ERβ expression is frequently associated with disease progression and may negatively impact the response to treatment [3]. Mechanistically, ERβ has been implicated in initiating the apoptotic cycle and influencing the cellular metabolism of malignant cells [4,5].

CRC remains a massive global health burden. In the United States, CRC is projected to account for 152,810 new cases in 2024, contributing to an estimated 53,010 deaths, making it the second leading cause of cancer-related mortality [6]. This incidence is unequally distributed, with men accounting for approximately 81,540 cases and women for 71,270 cases. A particularly alarming epidemiological trend is the continuous rise in CRC incidence and mortality rates among younger adults. Since the mid-1990s, the incidence rate has increased by 1% to 2% each year in individuals under the age of 55 [6]. CRC has now become the leading cause of cancer death in men under 50 and the second leading cause in women of the same age group. This shift reinforces the urgent need to identify robust molecular targets, such as ERβ, that may be involved in the pathogenesis of early-onset disease, especially since younger patients are often diagnosed with more advanced cancers due to delays in detection [7].

The strategy for mitigating the CRC burden is prevention and early detection, designed to intercept the disease during the adenoma-carcinoma sequence. Current US Preventive Services Task Force (USPSTF) guidelines recommend that screening commence for average-risk adults between the ages of 45 and 75. The decision to screen patients aged 76 to 85 should be individualized [8]. The imperative to detect CRC at the earliest possible stage, or ideally at the adenomatous polyp stage, is supported biologically by the observed loss of protective signaling molecules during this transition. The decline of ERβ expression during the adenoma-to-carcinoma progression suggests it may be a molecular event critical to the malignant transformation process.

Primary prevention of CRC focuses on modifying lifestyle and environmental factors that drive the adenoma–carcinoma sequence. Maintaining a healthy body weight, being physically active, limiting alcohol and red/processed meat, avoiding smoking, and controlling metabolic risk factors are each associated with reduced CRC incidence. In selected individuals, regular low-dose aspirin offers additional chemopreventive benefit [9]. Secondary prevention aims to detect and treat preclinical or recurrent CRC, primarily through endoscopic and stool-based screening. Colonoscopy, which allows direct visualization, biopsy, and immediate polypectomy throughout the colon, is considered the reference standard for both screening and surveillance [10]. Noninvasive stool tests, including fecal occult blood tests, fecal immunochemical tests, and multitarget stool DNA assays, also reduce CRC mortality when performed regularly [11]. Emerging blood-based “liquid biopsy” approaches measure circulating tumor DNA (ctDNA) or other tumor-derived components and can detect tumor-specific sequence or methylation changes associated with early CRC. Currently, they act as complementary tools rather than replacements for established population screening methods [12].

The incidence of CRC is lower, and prognosis is better in women than in men up to the age of menopause, whereas this sex difference diminishes in older women. Loss of ERβ expression appears to influence colorectal carcinogenesis, and ERβ may exert a protective, tumor-suppressive effect. In this meta-analysis, we evaluated studies that compared ERβ protein expression in CRC tissue with adjacent normal mucosa and in high-grade versus low-grade CRC. By integrating these data with clinicopathological outcomes, we aimed to clarify the prognostic value of ERβ and its potential as a therapeutic target in CRC.

## 2. Rationale and Aim of the Study

While preclinical studies have consistently linked loss of ERβ to increased proliferation and advanced disease [13], clinical findings often exhibit variability due to differences in cohorts, detection methodologies, and cut-off values [7,14]. A synthesized, quantitative assessment is necessary to confirm the clinical significance of ERβ expression status across varying stages of CRC. This study aimed to conduct a systematic review and meta-analysis to investigate and quantify the relationship between ERβ expression and CRC progression. Specifically, the study sought to determine the pooled odds ratio quantifying the difference in ERβ expression between tumor tissue and normal controls, and between early-stage disease and late-stage disease, thereby establishing its prognostic role and informing future therapeutic strategies.

## 3. Methods

### 3.1. Identification of Studies and Data Extraction

For this meta-analysis, comprehensive searches were conducted in the MEDLINE, PubMed, Embase, and Google Scholar databases up to 31 December 2025. The focus was on identifying English-language human studies, utilizing the following search text and/or Medical Subject Headings (MeSH) terms: “Colorectal cancer” OR “CRC” [All Fields] AND “ERβ”. A manual search was also conducted, reviewing the bibliographies of retrieved original studies, review articles, and published editorials. This meta-analysis was conducted in accordance with the Preferred Reporting Items for Systematic Reviews and Meta-Analyses (PRISMA) statement for interventions [15]. The PRISMA 2020 checklist is provided in the Supplementary Materials.

### 3.2. Selection Criteria—Primary Endpoints

Inclusion and exclusion criteria were established before initiating the study investigation. Eligible studies were incorporated into the meta-analysis if they were: a. published as complete articles with extractable data; b. written in English; and c. comparing CRC tissue with normal controls, simple adenomatous polyps with advanced adenomas, and early stages of CRC with late stages. Case–control studies comparing ERβ expression in CRC biopsy specimens with that in healthy controls were included.

Studies utilizing standard immunohistochemistry (IHC) or Western blot techniques with antibodies targeting ERβ, as well as PCR for RNA analysis, were included. Critically, only studies reporting results in terms of the percentage of moderate and/or strong positive staining were considered. Studies expressing results as mean ± SD of staining scores were excluded, as these metrics could not be effectively synthesized in a meta-analysis focused on Odds Ratios. The primary endpoint was defined as the resolution of the OR and 95% Confidence Interval (95% CI) comparing expression rates.

### 3.3. Statistical Analysis

Meta-analysis was performed using Comprehensive Meta-Analysis software (Version 4, Biostat Inc., Englewood, NJ, USA) [16]. The random-effects model was employed for calculating pooled ORs and 95% CIs, based on the assumption that the true effects varied across the studies due to inherent biological or methodological differences.

### 3.4. Heterogeneity, Sensitivity, and Publication Bias

Heterogeneity was assessed using the Cochran Q test and the I^2^ Inconsistency index. Heterogeneity was considered present if the Q-test *p* value was less than 0.10. I^2^ values of 25%, 50%, and 75% represented low, moderate, and high inconsistency, respectively.

Sensitivity testing involved systematically excluding individual studies and recalculating the overall meta-analysis outcome to ensure the stability and robustness of the primary result. Publication bias was analyzed using a funnel plot, complemented by Begg-Mazumdar and Egger statistics [17]. Comparison-adjusted funnel plots were also constructed to examine their symmetry.

## 4. Results

### 4.1. Study Selection and Descriptive Characteristics

**Figure 1:**
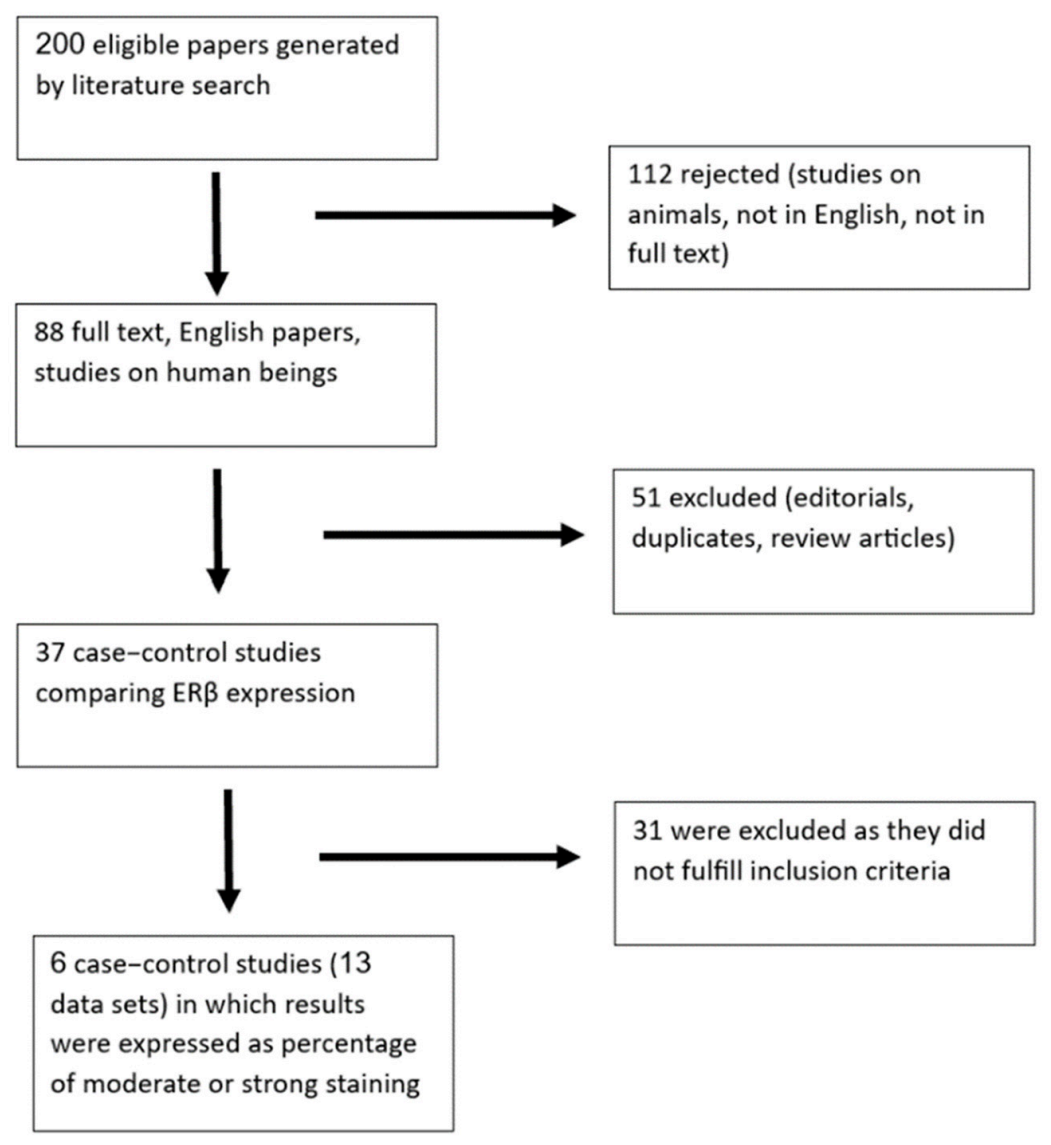
Flow chart of studies included in the meta-analysis.

The literature search revealed 200 eligible papers, from which six articles contributing 13 sub-studies (data sets) were selected for the final meta-analysis after rigorous application of the exclusion criteria (Figure 1). Reasons for exclusion included lack of full text or English language (112 papers), being editorials, reviews, or duplicates (51 papers), or failing to fulfill the inclusion criteria (31 papers). The included studies were published up to 31 December 2025, and originated from five countries: Germany, China, Serbia, Greece, and Brazil. These studies collectively involved 1183 patients with tumors (adenomatous polyps or CRC), compared to 1000 healthy controls or patients with early-stage adenomas or cancer.

### 4.2. Qualitative Findings of Included Studies—A Systematic Review [18,19,20,21,22,23]

All six studies consistently supported the conclusion that ERβ expression declines with CRC progression and correlates with poor outcomes.

**Konstantinopoulos PA et al. (2003)** [18] analyzed ERβ expression in CRC cases and corresponding normal colonic mucosa using IHC. ERβ was detected in a significantly lower proportion in tumor tissues compared to normal tissues (56 of 90 cases versus 85 of 90 cases, respectively). This reduction was more pronounced in poorly differentiated cancers compared to moderately differentiated ones (8 of 19 cases versus 30 of 48 cases), and in moderately differentiated CRC compared to well-differentiated CRC (30 of 48 versus 18 of 23). This confirmed that ERβ loss parallels the tumor’s dedifferentiation. Internal validity is enhanced by paired sample analysis and standardized IHC across various cell types. However, the study’s applicability and prognostic value are limited by its modest sample size, single-center design, and lack of standardized scoring.

**Rudolph A et al. (2012)** [19] evaluated the prognostic impact of ERβ expression in a large cohort of CRC patients. They found that the loss of ERβ expression (**the loss is detected** in 566 out of 1101 cases) was significantly associated with advanced tumor stage, greater tumor extent, and, notably, reduced disease-free and overall survival. ERβ expression was detected in 187 out of 426 stage III tumors compared to 125 out of 244 stage I tumors. The study’s large population-based cohort and rigorous statistical modeling provide strong evidence linking ERβ negativity to advanced disease stages and poorer outcomes. Nevertheless, the findings are limited by technical constraints such as tissue loss and unsuccessful staining, as well as potential selection bias arising from the observational design.

**Wang Y et al. (2015)** [20] analyzed both ERβ mRNA and protein levels in CRC tissues. ERβ was detected in a significantly lower proportion of tumor tissues than in adjacent normal tissues (117 of 203 versus 181 of 203). Furthermore, marked ERβ expression was lower in patients with lymph node metastasis compared to normal tissues (59 of 95 versus 86 of 95), highlighting its association with metastatic potential. Similarly, among CRC patients without metastasis compared to normal tissues, 58 of 108 versus 95 of 108 were ERβ positive, respectively. The study’s concurrent quantification of ERβ mRNA and protein in paired tissues confirms consistent downregulation and links reduced expression to metastasis and advanced disease. Conversely, its external validity is limited by a single-ethnicity cohort, a hospital-based design, and the absence of data regarding survival impact.

**Stevanato Filho P et al. (2018)** [21] investigated ERβ expression by immunohistochemistry in patients with adenomatous polyps or invasive CRC, with or without familial adenomatous polyposis (FAP). ERβ expression differed significantly between groups. ERβ expression was lower in FAP polyps compared to sporadic polyps. 23 out of 30 FAP samples showed ERβ expression, versus 30 out of 30 sporadic polyps. The transition from sporadic polyps to CRC was associated with a marked decrease in ERβ expression in 18 out of 30 cancer samples versus 30 out of 30 polyps. Advanced tumors (T3/T4) demonstrated greater ERβ loss than early-stage tumors (T1/T2), with 27 out of 43 and 16 out of 17 showing ERβ expression, respectively. Patients with lower 5-year survival expressed ERβ in 8 out of 17, and patients with higher 5-year survival in 32 out of 43. The study establishes ERβ loss as a key marker of tumor progression and survival by examining both hereditary and sporadic lesions across the adenoma–carcinoma sequence. Yet, the small sample size and single-center design may limit the statistical power of these findings.

**Babic A et al. (2021)** [22] analyzed ERβ in 101 CRC biopsies. ERβ expression was assessed by immunohistochemistry. ERβ expression was found in 6 of 51 early recurrence patients with disease-free survival (DFS) ≤ 24 months versus in 20 of 50 long-term survivors with DFS ≥ 48 months. The study’s use of a homogeneous high-risk cohort and uniform treatment protocols ensures strong internal consistency for its findings on early recurrence. Nevertheless, its retrospective, single-center design and small sample size may limit the statistical power of the results.

**Honma N et al. (2022)** [23] analyzed ERβ expression in CRC surgical specimens from postmenopausal Japanese women. ERβ expression was assessed by immunohistochemistry. In the left-sided colon of younger patients under 70, cancerous tissues demonstrated a significantly lower ERβ positivity rate compared to their non-cancerous tissue, 10 out of 23 versus 16 out of 19, respectively. Combining hormone measurements with molecular data provides a strong methodological and mechanistic framework. However, the modest sample size, specific ethnic focus, and lack of survival data limit its broader applicability.

### 4.3. Meta-Analysis Outcomes

**Figure 2:**
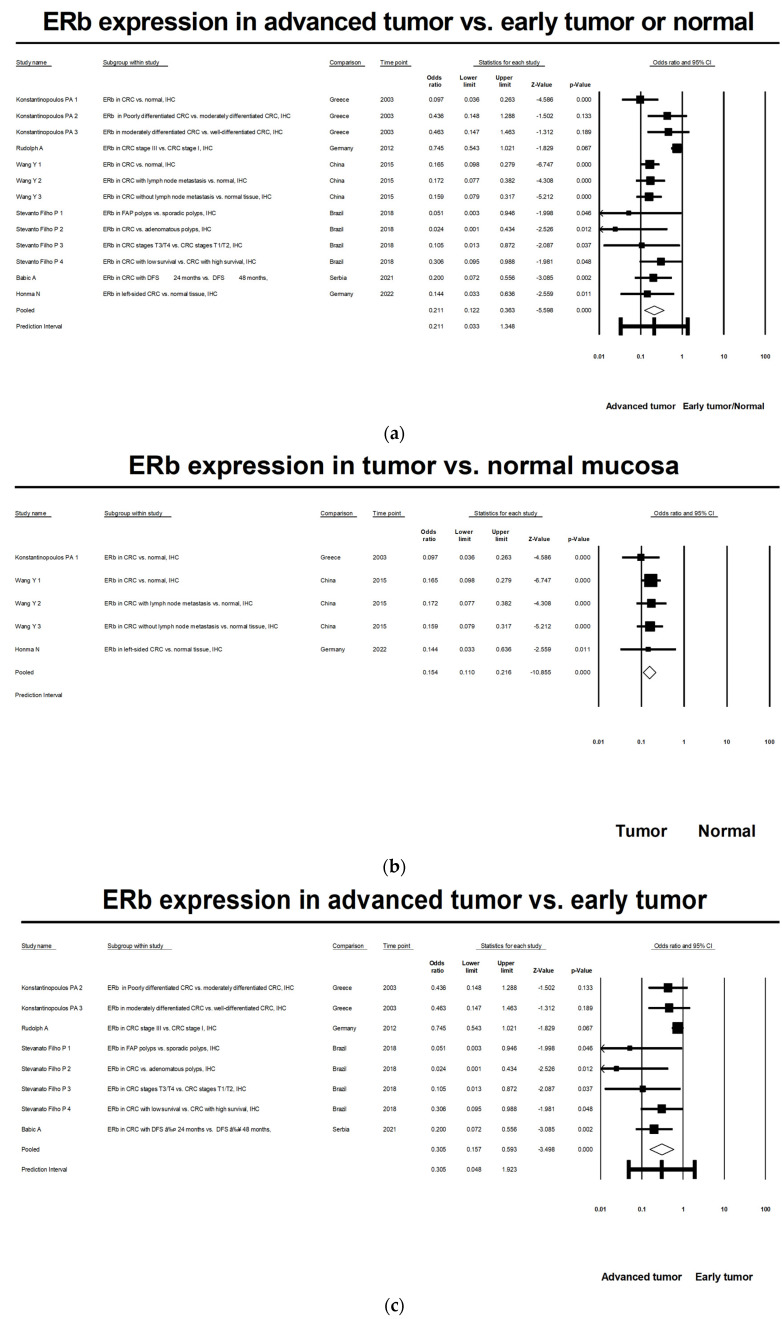
(**a**) Forest plot illustrating OR and 95%CI for ERβ expression in late-stage tumor patients vs. early-stage tumors, and normal mucosa. (**b**) Forest plot illustrating OR and 95%CI for ERβ expression in tumor patients vs. healthy controls. (**c**) Forest plot illustrating OR and 95%CI for ERβ expression in early-stage CRC and simple adenomatous polyps vs. late-stage CRC and advanced adenomas.

**Figure 3:**
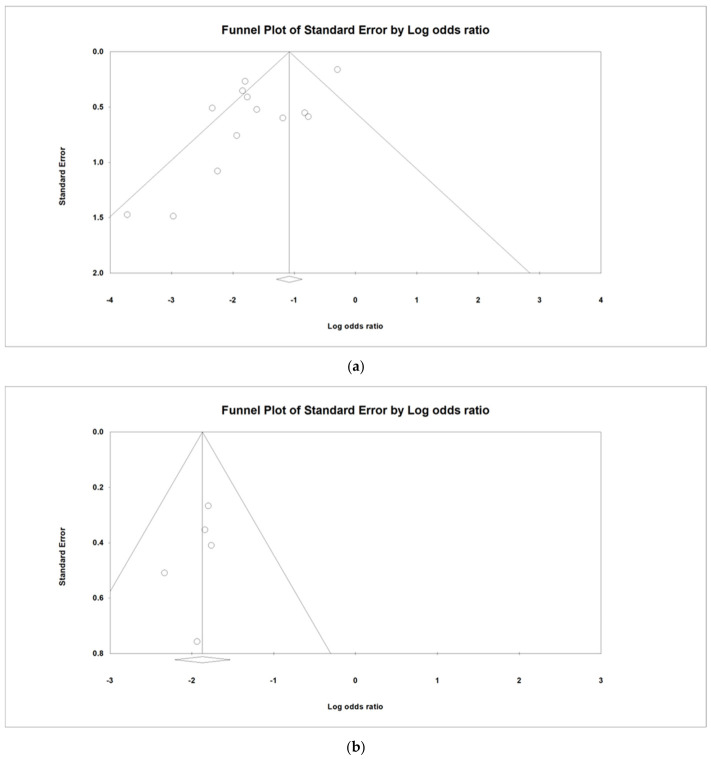
(**a**) Funnel plot for publication bias of all the studies. (**b**) Funnel plot for publication bias of studies comparing tumors and normal mucosa.

**Figure 4:**
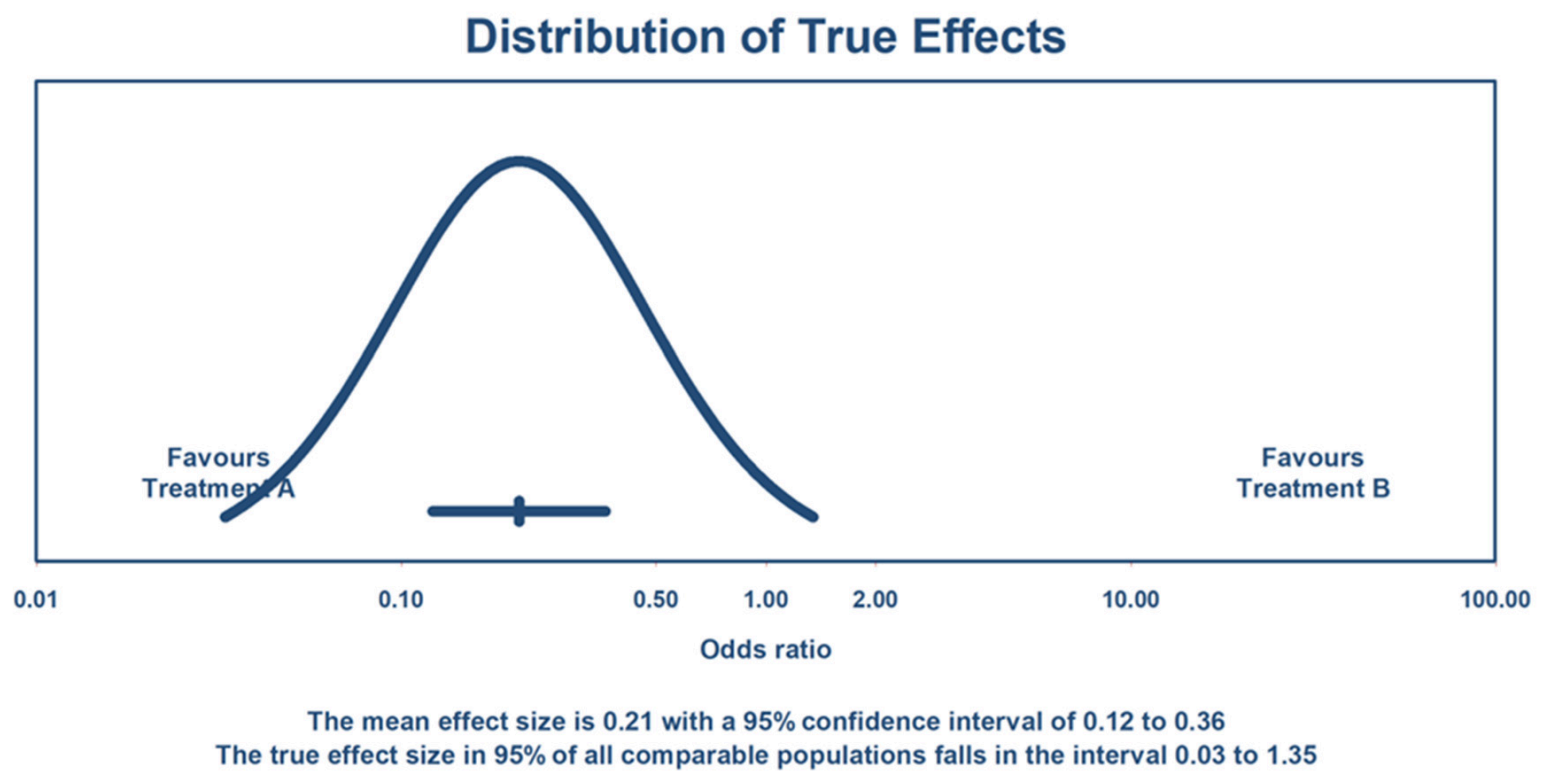
Range of true effects.

We included in our meta-analysis six articles and 13 sub-studies (data sets) comparing cancer with normal tissue, early stages of CRC with late stages, and simple with advanced adenomatous polyps studies, published up to 31 December 2025 [18,19,20,21,22,23]. Altogether, there were 1183 patients with tumors (adenomatous polyps or CRC), compared to 1000 healthy controls or patients with early-stage adenomas or cancer, available for ERβ expression comparison (Figure 2a). Of these, 607 (51.31%) and 764 (76.40%) were positive, respectively. ERβ expression could be calculated for 519 CRC patients (all stages) compared only with 515 healthy controls (Figure 2b) and for 664 patients with advanced-stage CRC versus 485 patients with early-stage CRC (Figure 2c). For the latter, 300 (57.8%) versus 463 (89.90%), and 307 (46.23%) versus 301 (62.06%) were positive for ERβ staining, respectively (*p* < 0.001). The mean effect size of ERβ expression is 0.211, 95% CI: 0.122 to 0.363, *p* < 0.0001. The mean effect size in the universe of comparable studies could fall anywhere in this interval. The Z-value tests the null hypothesis that the mean effect size is 1.000 and is −5.598 with *p* < 0.001. Using a criterion alpha of 0.050, we reject the null hypothesis and conclude that in the universe of populations comparable to those in the analysis, the mean effect size is not precisely 1.000. The relevant funnel plot (Figure 3a) indicates a mild effect from small studies but suggests no significant publication bias (*p* > 0.05), **with only 13 data points; Begg-Mazumdar and Egger tests for publication bias have limited statistical power**. The funnel plot of studies comparing only CRC with normal mucosa has no publication bias (Figure 3b). The Q-statistic provides a test of the null hypothesis that all studies in the analysis share a common effect size. If all studies shared the same true effect size, the expected value of Q would be equal to the degrees of freedom (the number of studies minus 1). The Q-value is 53.395 with 12 degrees of freedom and *p* < 0.001. Using a criterion alpha of 0.100, we can reject the null hypothesis that the true effect size is the same in all these studies. The I-26291squared statistic is 78%, indicating that approximately 78% of the variance in observed effects is due to variance in true effects rather than sampling error. **The prediction interval (0.033–1.348) includes OR = 1, indicating that in some plausible populations the true effect could be null or reversed, and that** the true effects are normally distributed (in log units) (Figure 4). We measured sensitivity by excluding individual studies and recalculating the overall meta-analysis outcome. This process was repeated for each of the studies. Deviations from the primary result were not significant. Calculating studies separately with OR up to and above the median OR of 0.165, respectively, all in the same direction (higher in normal mucosa and in early stages of the tumor tissues) and within the prediction interval: from OR 0.024 to 0.159, **OR 0.124**, 95%CI 0.075 to 0.204, *p* < 0.0001; from OR 0.172 to 0.745, **OR 0.358**, 95%CI 0.051 to 4242.493, *p* < 0.001. Calculating studies separately according to the number of patients (below and above the median of 48 patients for a study), again, all the results were within the predictive value: from 17 to 43 patients, **OR 0.207**, 95%CI 0.102 to 0.422, *p* < 0.0001; from 51 to 426 patients, **OR 0.209**, 95%CI 0.093 to 0.457, *p* < 0.0001.

## 5. Discussion

### 5.1. Quantifying ERβ Loss as a Defining Event in CRC Progression

A decrease in the expression of ERβ in CRC tissue in both men and women may indicate its role as a tumor suppressor gene. This may come into action mainly in women, where circulating blood estrogen is significantly higher than in men. This systematic review and meta-analysis provide compelling and quantitative evidence that the loss of ERβ expression is a defining molecular event in the progression of CRC. The highly significant pooled OR of 0.211 confirms the substantial magnitude of the protective effect associated with ERβ expression. This finding is consistent across the included heterogeneous patient cohorts, validating the premise, established in preclinical models, that ERβ acts as a fundamental tumor suppressor in the colon [7]. The clinical relevance of this loss is substantiated by the consistent association between reduced ERβ expression and markers of aggressive disease, including advanced tumor stage, lymph node metastasis, tumor dedifferentiation, reduced overall survival, and increased risk of early recurrence. Consequently, ERβ status emerges as a clinically valuable prognostic biomarker.

### 5.2. Critical Appraisal of Included Data and Sources of Heterogeneity

While the aggregated data strongly support a universal protective role for ERβ, the quantitative analysis revealed high inconsistency (I^2^ = 78%), necessitating a critical appraisal of the contributing factors. The meta-analysis benefits from incorporating several robust studies, notably the large prospective cohort reported by Rudolph et al. (N = 1101), which confirmed the prognostic significance of ERβ negativity for overall and disease-free survival [19]. Furthermore, the inclusion of data spanning the entire spectrum of colorectal carcinogenesis—from simple adenomatous polyps to advanced, metastatic carcinoma, and focused analyses on specific outcomes like recurrence [22] and familial polyposis [21] strengthens the conclusion that ERβ loss is integral to malignant advancement. However, the identified heterogeneity primarily stems from methodological variability and biological factors.

### 5.3. Molecular Mechanisms of ERβ Tumor Suppression

The protective role of ERβ in CRC pathogenesis is attributed to its dual functions: the suppression of cellular proliferation (carcinogenesis) and the inhibition of metastasis and invasion. The reduction in ERβ expression in cancer tissue is therefore not merely a consequence of abnormal differentiation, but likely a mechanism exploited by cancer cells to promote growth and spread [3,4,5,7,13,24,25,26,27,28]. Loss of ERβ in ERβ knockout mice results in hyperproliferation of the colonic mucosa, impaired apoptosis, and disturbances in tight junction integrity and mucin production. ERβ acts as a dominant regulator, often exerting anti-proliferative effects that oppose the growth-promoting signals mediated by ERα when the two receptors are co-expressed. Furthermore, ERβ is capable of standalone tumor-suppressive actions, even in the absence of ERα. Specifically, ERβ drives pro-apoptotic signaling, partly by activating pathways such as the p38/MAPK pathway, thereby compelling malignant cells toward programmed death. Activation of ERβ has also been shown to induce transcriptome changes that enhance anti-tumorigenic capabilities, including regulating the cell cycle, promoting differentiation, and increasing DNA-repair capacity [24]. A crucial finding is ERβ’s ability to significantly downregulate inflammatory pathways, including the downstream networks of Interleukin-6 (IL-6). This anti-inflammatory action is biologically critical, given the well-established link between chronic inflammation (e.g., in inflammatory bowel disease) and colitis-associated carcinogenesis [28].

Thus, ERβ limits CRC cell proliferation by restraining key cell cycle and oncogenic pathways, and increasing pro-apoptotic signals. Experimental overexpression of ERβ in colon cancer cells reduces cyclin D1/E and β-catenin signaling, increases p21 and other p53 pathway mediators, and induces G1–S arrest and apoptosis, consistent with a tumor suppressive role in early carcinogenesis.

### 5.4. Prevention of Metastasis and Invasion

The finding that ERβ expression is specifically reduced in cases with lymph node metastasis suggests a distinct role in preventing tumor spread. This role is molecularly explained by the newly characterized ERβ-miR-205-PROX1 signaling axis [24]. The process begins with ERβ activation, which upregulates the expression of microRNA-205 (miR-205). MiR-205 then acts as a crucial tumor suppressor by directly targeting and silencing the 3’{UTR} of the PROX1 oncogene mRNA. PROX1 is known to increase cell invasiveness and metastatic potential. Therefore, the progressive loss of ERβ observed in advanced tumor stages leads to the corresponding loss of miR-205, which, in turn, de-represses PROX1, initiating and facilitating the invasion and metastatic processes. This complex regulatory cascade confirms ERβ’s protective role extends beyond primary tumor growth inhibition to actively suppressing the spread of the disease. In addition, ERβ suppresses the expression of several EMT and metastasis-related genes, such as β-catenin, Slug and Twist [29].

CRC patients with high ERβ expression combined with absent ERα expression show markedly prolonged overall survival and disease-free survival. In contrast, low ERβ expression is associated with an increased risk of local tumor recurrence [14]. It has also been found that ERβ status in CRC is linked to a distinct epigenetic landscape, involving hundreds of CpG sites across key oncogenic pathways. ERβ-associated methylation changes in genes related to proliferation, invasion, and DNA repair (including HK1, CD36, LRP5, and MLH1) suggest that preserved ERβ expression may help sustain a more favorable epigenetic profile, thereby contributing to a better prognosis [30].

In this meta-analysis, there is robust statistical evidence supporting the progressive downregulation of ERβ throughout the sequence of colorectal carcinogenesis. The strength of this association is visually and quantitatively demonstrated in Figure 2a–c, which reveals a consistent pattern of reduced expression across geographically diverse populations. The pooled odds ratio of 0.211 indicates a strong inverse correlation between ERβ presence and malignancy. Notably, the stratification of data reveals that this loss of expression is not merely binary (cancer versus normal) but gradational. The significant reduction observed when comparing advanced-stage to early-stage disease (Figure 2c) aligns with the histological findings of Rudolph et al. and Stevanato Filho et al., suggesting that ERβ silencing is a continuous event driving tumor progression and dedifferentiation [19,21].

### 5.5. Clinical Implications and Future Therapeutic Strategies

The identification of ERβ status as a reliable prognostic factor has immediate clinical implications. It can potentially be integrated into prognostic panels to better stratify patients, particularly those with high-risk stage III (Duke’s C) CRC, where decreased ERβ expression is linked to early recurrence. Such patients may benefit from more aggressive adjuvant therapy or intensified surveillance schedules. Furthermore, the robust evidence supporting ERβ’s protective function points toward targeted activation as a viable chemopreventive or therapeutic approach [24]. The development and utilization of ERβ-selectiveLine 21 agonists (SERBAs) or natural compounds such as phytoestrogens (e.g., silibinin) represent a promising therapeutic route [24]. These agents aim to enhance ERβ signaling in the colon epithelium to inhibit polyp formation and progression, particularly in high-risk patients such as those with familial adenomatous polyposis (FAP) or Lynch syndrome. Preclinical studies have shown that targeted ERβ activation can reduce tumor growth and suppress angiogenesis. Further clinical trials are warranted to fully determine the utility of ERβ activation in the prevention and adjuvant treatment of CRC. Even in tumors where ERβ expression is completely lost, the specific molecular pathways can still be targeted. For instance, miR-205 replacement therapy could be explored as a direct means to suppress PROX1 expression and combat metastasis, offering a novel intervention strategy independent of the receptor status.

## 6. Limitations

Potential limitations should be considered in this review: (1) Only English-language literature was included, potentially leading to selection bias. (2) The meta-analysis included studies that used different detection methods (IHC vs. PCR) and cut-off values for positivity, which contributed significantly to the observed high heterogeneity. (3) Our data involved patients from only five countries, indicating a possibility for variation in results across other geographical areas or ethnic groups that have not yet been studied.

## 7. Methodological Limitations

The included studies employed different methods (IHC, Western blot, PCR). IHC, while standard, is subject to variation based on the specific antibody clone, tissue fixation methods, and inherent subjectivity in determining the percentage and intensity cut-offs defining “positive” expression. In addition, the decision to exclude studies reporting results as mean ± SD (staining scores) ensured the mathematical feasibility of the meta-analysis but may have led to the exclusion of relevant biological data.

### Biological Heterogeneity

The cohorts studied involved patients across five different geographical areas and represented diverse tumor characteristics (e.g., sporadic vs. FAP, varying stages, and specific hormonal contexts like postmenopausal women). Cancer exhibits significant intratumoral heterogeneity, where distinct cell populations within the same tumor specimen possess different molecular profiles. The expression level and functional status of ERβ can vary dynamically within a tumor, influencing the reported percentage of positive staining and contributing to the observed high inconsistency [24,25,26,27]. Despite the high I^2^ value, the overall pooled effect was robust and maintained significance across all subgroups and sensitivity analyses, suggesting that the fundamental biological mechanism—loss of ERβ being detrimental—is universally present, even if the precise clinical consequences vary across populations and methodologies. In addition, achieving similar results as a previous meta-analysis **(in an additional 6 studies)** is a strength, considering better methods and accuracy of the laboratories in the last decade [31].

## 8. Conclusions

This systematic review and meta-analysis confirmed that ERβ expression is significantly lower in CRC tissues compared to healthy controls, and that its progressive loss is strongly associated with advanced disease stages and poor prognosis. This protective effect is mediated by the dual roles of ERβ in suppressing cellular proliferation, promoting apoptosis, and critically inhibiting metastatic potential through the ERβ-miR-205-PROX1 signaling axis. ERβ status represents a valuable prognostic marker and a promising molecular target for novel chemopreventive and therapeutic interventions in CRC management.

## Data Availability

No new data were created or analyzed in this study. Data sharing is not applicable to this article.

## Supplementary Materials

The following supporting information can be downloaded at: https://www.mdpi.com/article/10.3390/jcm15155924/s1. Reference [32] is cited in Supplementary Materials.

## Abbreviations

ERβ	Estrogen Receptor Beta
CRC	Colorectal Cancer
or	Odds Ratio
CI	Confidence Interval
